# Prevalence and risk factors of Japanese encephalitis virus (JEV) in livestock and companion animal in high-risk areas in Malaysia

**DOI:** 10.1007/s11250-017-1490-6

**Published:** 2017-12-14

**Authors:** Kiven Kumar, Siti Suri Arshad, Gayathri Thevi Selvarajah, Jalila Abu, Ooi Peck Toung, Yusuf Abba, Faruku Bande, A. R. Yasmin, Reuben Sharma, Bee Lee Ong, Anisah Abdul Rasid, Norsuzana Hashim, Amira Peli, E. P. Heshini, Ahmad Khusaini Mohd Kharip Shah

**Affiliations:** 1 Department of Veterinary Pathology and Microbiology, Faculty of Veterinary Medicine, Universiti Putra Malaysia, 43400 UPM Serdang, Selangor Malaysia; 2 Department of Veterinary Clinical Studies, Faculty of Veterinary Medicine, Universiti Putra Malaysia, 43400 UPM Serdang, Selangor Malaysia; 3 Department of Veterinary Laboratory Diagnostics, Faculty of Veterinary Medicine, Universiti Putra Malaysia, 43400 UPM Serdang, Selangor Malaysia; 4 Department of Veterinary Pathology and Microbiology, Faculty of Veterinary Medicine, City Campus, Universiti Malaysia Kelantan, Kota Bharu, Kelantan Malaysia; 5 Department of Conservation of Biodiversity of Wildlife and National Park Malaysia, Jalan Cheras, 56100 Kuala Lumpur, Malaysia

**Keywords:** Japanese encephalitis, Malaysia, Risk factor, ELISA, Prevalence

## Abstract

Japanese encephalitis (JE) is vector-borne zoonotic disease which causes encephalitis in humans and horses. Clinical signs for Japanese encephalitis virus (JEV) infection are not clearly evident in the majority of affected animals. In Malaysia, information on the prevalence of JEV infection has not been established. Thus, a cross-sectional study was conducted during two periods, December 2015 to January 2016 and March to August in 2016, to determine the prevalence and risk factors in JEV infections among animals and birds in Peninsular Malaysia. Serum samples were harvested from the 416 samples which were collected from the dogs, cats, water birds, village chicken, jungle fowls, long-tailed macaques, domestic pigs, and cattle in the states of Selangor, Perak, Perlis, Kelantan, and Pahang. The serum samples were screened for JEV antibodies by commercial IgG ELISA kits. A questionnaire was also distributed to obtain information on the animals, birds, and the environmental factors of sampling areas. The results showed that dogs had the highest seropositive rate of 80% (95% CI: ± 11.69) followed by pigs at 44.4% (95% CI: ± 1.715), cattle at 32.2% (95% CI: ± 1.058), birds at 28.9% (95% CI: ± 5.757), cats at 15.6% (95% CI: ± 7.38), and monkeys at 14.3% (95% CI: ± 1.882). The study also showed that JEV seropositivity was high in young animals and in areas where mosquito vectors and migrating birds were prevalent.

## Introduction

Japanese encephalitis virus (JEV) is vector-borne virus that causes Japanese encephalitis (JE), a severe zoonotic neurological disease. The virus is an enveloped single-stranded positive sense RNA virus that is endemic in Asian countries. JE is fatal to almost one third of affected human population. It is estimated that approximately three billion people worldwide are at risk of acquiring JE with 50,000–175,000 cases (Impoinvil et al. 2013, pp. 1–10; Van den Hurk et al. 2009, pp. 17–35; Campbell et al. 2011, pp. 766–774) with 10,000 to 15,000 deaths annually (Erlanger et al. 2009, pp. 1–7; Solomon, 2004, pp. 1803–1804; Solomon, 2006, pp. 869–871). However, some studies showed that although the mortality rate can be as high as 30% (Impoinvil et al. 2013, pp.1–10; Van den Hurk et al. 2009, pp. 17–35), < 4% of infected people will develop encephalitis during a JEV epidemic (Ricklin et al. 2016, pp. 10832). Transmission of JE involves a mosquito vector, particularly the *Culex tritaeniorhynchus*. Birds are reservoirs for JEV. Pigs and ardeidae birds are the principal amplifying hosts and they play important roles in the maintenance and transmission of the disease (Monath, 1988, pp. 139; Mackenzie et al. 2006, pp. 201–268). Animals that can be infected with JEV include horses, dogs, chickens, ducks, cattle, cats, bats, snakes, frogs, sheep, goats, wild boars, monkeys, raccoons, water buffalos, and birds (Bhattacharya and Basu 2014, pp. 32–37). Among the risk factors to the transmission of JEV are birds migrating from endemic countries, wind-blown mosquitoes, pig farming close to paddy fields, climate change, stagnant water, and introduction of animals from JEV-infected areas (Sulkin et al. 1970, pp. 77–87). Pet dogs and cats with JEV seroprevalence and are in close contact with owners increase the risk of humans and another animals acquiring the infection (Shimoda et al. 2011, pp. 241–1242). Besides this, birds have also been considered as reservoirs of JEV. There are no trace data of risk factors reported in cats, dogs, monkey, cattle, and birds in endemic countries like Malaysia. In Malaysia, most of the studies on JEV infection were conducted on the mosquitoes (Vythilingam et al. 1997, pp. 257–262; Simpson et al. 1970, pp. 503–510) and humans (Ooi et al. 2008, pp. 458–468; Cardosa et al. 1995, pp. 272–275) only. Thus, this study was undertaken to determine the JEV antibody and the risk factors in cats, dogs, monkeys, cattle, pigs, and birds in risk areas of Peninsular Malaysia.

## Materials and methods

### Ethical approval

This study was approved by the institutional animal care and use committee of Universiti Putra Malaysia with reference number UPM/IACUC/AUP. ROOS/2015.

### Study areas

**Fig. 1:**
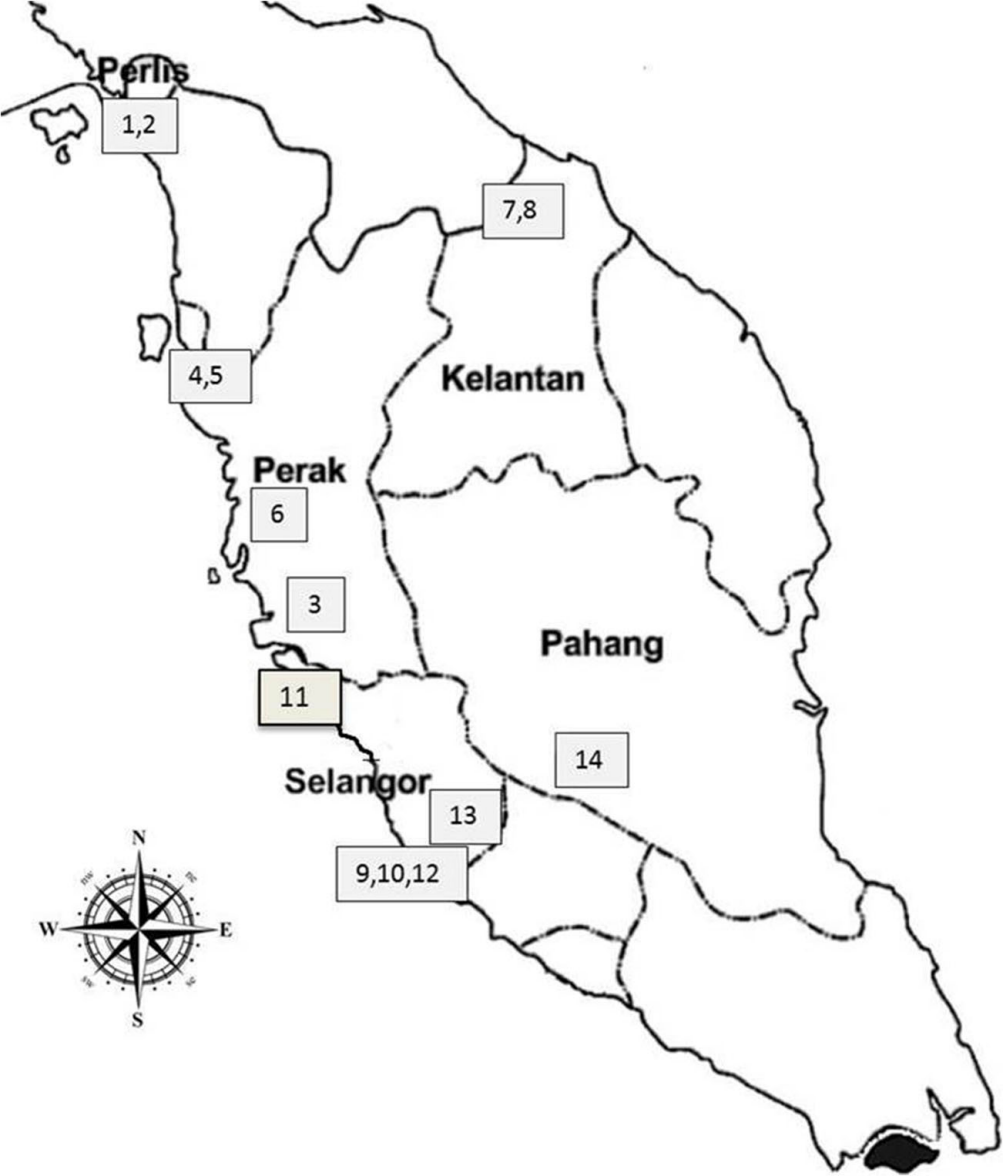
Geographic location of sample collection in Peninsular Malaysia

The study was carried out in Peninsular Malaysia. Peninsular Malaysia is located between 1° N and 7° N of the equator covering a distance of 124,450 km^2^. The study focused on high-risk areas like paddy cultivation areas, migratory bird landing areas, nearness to pig farms, high water bodies, high population of bats, forest areas, and construction areas which can contribute to JEV transmission in the following Peninsular Malaysia states; Perak, Selangor, Perlis, Kelantan, and Pahang. The most important risk areas in noticed in Perak, Kelantan, Perlis, Pahang, and Selangor are paddy fields, migratory bird landing areas, plantation areas, construction areas, and pig farms. The samples were collected from (I) Perak—Tapah (3) (4.1977° N, 101.2615 E), Parit Buntar (4) (5.1187° N, 100.4880° E), Tanjong Piandang (5) (5.0760° N, 100.3918° E), and Gopeng (6) (4.4717° N, 101.1654° N); (II) Selangor—Serdang (9) (3.0220° N, 101.7055° E), Putrajaya (10) (2.9264° N, 101.6964° E), Sabak Bernam (11) (3.6758° N, 100.9900° E), Jenderam Hulu (12) (2.8535° N, 101.74425° E), and Kepong (13) (3.2140° N, 101.6356° E); (III) Perlis—Kangar (1) (6.4406° N, 100.1984° E) and Arau (2) (6.4297° N, 100.2698° E); (IV) Kelantan—Kuala Krai (7) (5.5308° N, 102.2019° E) and Machang (8) (5.7679° N, 102.2154° E); and (V) Pahang—Temerloh (14) (3.4486° N, 102.4163° E). Figure 1: Map of Malaysia showing the geographic locations of sampling sites in Peninsular Malaysia.

### Study design

A cross-sectional survey was carried out to determine the seroprevalence and risk factors of JEV in animals and avian species in Peninsular Malaysia. Sample collection was conducted between December (2015) to February in 2016 and March to August in 2016. Overall, 416 samples were collected from dogs, cats, water birds, village chickens, jungle fowl, long-tailed macaque, domestic pigs, and cattle. Blood samples were collected for serum separation in a total of 45 birds from Perak, Selangor, and Putrajaya. Cat samples were collected from shelters and domestic cats from Ipoh, Perak, Putrajaya, Selangor, and University Veterinary Hospital—Universiti Putra Malaysia (UVH, UPM), respectively. Ninety samples were collected from cat shelters and domestic cats from Perak and Selangor. However, only 45 dog samples were collected from shelters in Ipoh, Perak, and Selangor. A total of 56 serum samples from long-tailed macaque (*Macaca fascicularis*) were collected from three different states: Pahang, Kelantan, and Perlis. Pig samples were collected from Tapah (Perak) 45 and Sepang (Selangor) 45, and all cattle samples (*n* = 90) were collected from Sabak Bernam and Serdang areas under Selangor state.

### Samples processing

Plain blood tube (3 ml) was used to collect blood from the jugular vein of each animal and birds. The serum was collected and centrifuged at 4000 ×*g* for 10 min. The serum was collected and stored at − 20 °C until further use. All samples used in this study were collected by the attending veterinary physician.

### Serological analysis

Serological detection of antibodies to JEV was performed using a commercially available qualitative ELISA kit (Qualitative Chicken Japanese Encephalitis IgG Antibody) (JE-IgG) (MyBiosource, USA; specificities 100%, sensitivities 98.9%) for chicken and water birds; Qualitative Canine Japanese Encephalitis IgG Antibody (JE-IgG) (MyBiosource, USA; specificities 100%, sensitivities 98.9%) for dogs; Cat (JE-IgG) Elisa kit (SunRed Biotechnology, Shanghai; specificities 100%, sensitivities 99.9%) for cats; Bovine (JE-IgG) Elisa kit (SunRed Biotechnology, Shanghai; specificities 100%, sensitivities 99.9%) for cattle; Porcine (JE-IgG) Elisa kit (SunRed Biotechnology, Shanghai; specificities 100%, sensitivities 99.9%) and quantitative ELISA namely the Monkey Japanese Encephalitis Antibody IgG (JEAb-IgG), (MyBiosource, USA; specificities 100%, sensitivities 98.9%) for monkeys. All procedures were carried out according to the manufacturer’s protocol. Samples were duplicated and microplates were read at the wavelength of 450 nm within 15 min of adding the stop solution. The optical density (OD) was read using a TECAN infinite microplate reader (M200, Switzerland) with Magellan v.6.5 software (Austria). The test is validated if the mean values of negative control O.D (O.D_NC_) are less or equal to 0.15 and the mean value of positive control O.D (O.D_PC_) is more or equal to 1.00. For result interpretation, the cut off value was calculated using the following formula:

$$ \mathrm{CUTT}\ \mathrm{OFF}=\mathrm{O}.{\mathrm{D}}_{\mathrm{NC}}+(0.15) $$

- Negative judgment: if the OD value < CUT OFF, the sample JEV-IgG negative.
- Positive judgment: if the OD value ≥ CUT OFF, the sample JEV-IgG positive.

### Determination of the cut off value for quantitative Elisa

To determine the antibody concentration of the samples, the standard curve was constructed by plotting mean absorbance for each standard (0, 0.5, 1.0, 2.5, 5.0, 10 μg/ml) on the *x*-axis against the concentration on the *y*-axis. Then, the best fit curve was drawn trough the points on the graph using CurveExpert 1.4 giving a significant correlation coefficient (*R* ^2^ > 0.9868) and *y* = 1 / (*a* + *bx* ^c^). The positive samples were range between 0.5 and 10 μg/ml according to the manufacturer’s protocol.

### Collection of data collection for JEV “risk factor” survey

A 13-questionaire was distributed to the farmers in order to collect and identify the risk factors related with JEV seropositive. The survey questions were collected from farm owner the following: (a) Individual animal or bird: age, health status (healthy vs. sick—was diagnosed based on clinical records and general physical examination), source of animals or birds (same areas, same district, same farms, same state, or imported from epidemic areas) and breed types (animals or birds originate from Malaysia or were imported from another country), ownership of animals or birds (shelter, own or none), and presence of mosquitoes in sample collected areas (present or absent). (b) Environment factor: stagnant water (types of stagnant water are ditches or ponds within 100 to 500 m), paddy cultivation (within 200 to 500 m), presence of ardeid birds (within 1 to 2-km location of bird landing areas), locality (urban, forest, or rural was determined by combined population of 10,000 or more), state (Perak, Selangor, Kelantan, Perlis, or Pahang), and nature of state located (Center-Selangor, North-Perak, Perlis, East-Kelantan, Pahang).

### Data management and statistical analysis

All analyses were performed using the IBM SPSS Statistics V22.0 (IBM, USA). Prevalence was expressed as a percentage and calculated by dividing the number of positive samples by the total number of animals tested. Chi-square analysis (*χ* ^2^ test is not appropriate one of expected cell value was less than 5, Fisher’s exact test was used at *α* = 0.05) was used to determine the univariate association between each of the putative risk factors with JEV. Similarly, the odd ratio (OR) and its 95% confidence interval (CI) were calculated at a significance level of *α* = 0.05.

## Results

Based on the seroprevalence data obtained, dog had the highest prevalence (80%) followed by pig (44.4%), cattle (32.2%), and bird (28.9%). Cat and monkey both had the lowest prevalence among all animals sampled (Table 1).

**Table 1:** Seroprevalence of Japanese encephalitis virus (JEV) in animals and birds

Species	Seroprevalence (%)
Dogs	80.0
Pigs	44.4
Cattle	32.2
Birds	28.9
Cats	14.4
Monkeys	14.3

The individual risk factor assessment of JEV in cats showed significant influence (*P* > 0.05) of age, locality, breed, and source of cats to JEV exposure in these species. Other factors such as gender, health status, stagnant water, place, nature, and owner all did not show any significant influence on JEV (Table 2). Breed of cat showed a strong association with JEV. Imported breed of cats was nine times more likely to contract JEV compared to local breeds. The source of cat also showed significant (*P* < 0.05) seropositivity as cats from the same areas showed two times more seropositive rate than those from the same district.

**Table 2:** Risk factors for Japanese encephalitis virus (JEV) seropositivity in cats

Risk factors		*n*	JEV Ab^−^ (%)	JEV Ab^+^ (%)	OR	95% CI	*P* value
Cat (*n* = 90)							
Source	Same area Same district	58 32	79.3 96.9	20.7 3.1	1.545 0.191	1.214–1.967 0.029–1.281	*P* = 0.023 Ref
Gender	Male Female	47 43	83.7 87.2	16.3 12.8	1.152 0.867	0.66–2.010 0.465–1.617	*P* > 0.05 Ref
Age	Young Adult	22 68	54.5 95.6	45.5 4.4	4.936 0.273	0.101–0.741 2.711–8.985	*P* = 0.000 Ref
Breed	Local Import	85 5	88.2 40.0	11.8 60.0	0.790 8.885	0.585–1.066 1.640–48.146	*P* = 0.003 Ref
Healthy status	Sick Healthy	50 40	86.0 85.0	14.0 15.0	0.964 1.045	0.561–1.656 0.552–1.980	*P* > 0.05 Ref
Owner	Shelter Personal	45 45	82.2 88.9	17.8 11.1	1.281 0.740	0.786–2.087 0.360–1.522	*P* > 0.05 Ref
Collection area							
Locality	Selangor Perak	72 18	88.9 72.2	11.1 27.8	0.740 2.278	0.476–1.151 0.976–5.317	*P* > 0.05 Ref
	Urban Rural	70 20	90.0 70.0	10.0 30.0	0.658 2.538	0.394–1.101 1.194–5.398	*P* = 0.025 Ref
Stagnant water	Yes No	16 74	75.0 87.8	25.0 12.2	1.974 0.820	0.751–5.193 0.564–1.193	*P* > 0.05 Ref
Paddy field	Yes No	- 90	NA 85.5	NA 14.4	NA NA	NA NA	Ref Ref
Ardeid birds (presence)	Yes No	16 74	75.0 87.8	25.0 12.2	1.974 0.820	0.751–5.193 0.564–1.193	*P* > 0.05 Ref
Mosquitoes (presence)	Yes No	90 –	85.6 NA	14.4 NA	NA NA	NA NA	Ref Ref

Ab^−^ = antibody-positive; Ab^+^; OR = odds ratio; CI = confidence interval, NA = not applicable, Ref = reference category

In the dog, only the source of dog was the factor that significantly (*P* < 0.05) influenced JEV exposure, while all other factors evaluated did not have an influence on JEV exposure (Table 3).

**Table 3:** Risk factors for Japanese encephalitis virus (JEV) seropositivity in dogs

Risk factors		*n*	JEV Ab^−^ (%)	JEV Ab^+^ (%)	OR	95% CI	*P* value
Dog (*n* = 45)							
Source	Same area Same district	9 36	55.6 11.1	44.4 88.9	0.2 2.0	0.067–0.597 0.955–4.190	*P* = 0.003 Ref
Gender	Male Female	34 11	18.2 20.6	81.8 79.4	1.125 0.964	0.293–4.326 0.648–1.434	*P* > 0.05 Ref
Age	Young Adult	5 40	20.0 20.0	80.0 80.0	1.0 1.0	0.772–1.295 0.127–7.893	*P* > 0.05 Ref
Breed	Local Import	32 13	21.9 15.4	78.1 84.6	0.893 1.375	0.592–1.347 0.368–5.136	*P* > 0.05 Ref
Healthy status	Sick Healthy	7 38	28.6 18.4	71.4 81.6	0.625 1.107	0.144–2.713 0.762–1.608	*P* > 0.05 Ref
Owner	Shelter Personal	45 –	20.0 NA	80.0 NA	NA NA	NA NA	Ref Ref
Collection area							
Locality	Selangor Perak	23 22	30.4 9.1	69.6 90.9	0.571 2.5	0.711–8.784 0.345–0.947	*P* > 0.05 Ref
	Urban Rural	23 22	30.4 9.1	69.6 90.9	0.571 2.5	0.345–0.947 0.711–8.784	*P* > 0.05 Ref
Stagnant water	Yes No	22 23	9.1 31.4	90.9 69.6	2.5 0.571	0.711–8.784 0.345–0.947	*P* > 0.05 Ref
Paddy field	Yes No	– 45	NA 20.0	NA 80.0	NA NA	NA NA	Ref Ref
Ardeid birds (presence)	Yes No	22 23	9.1 30.4	90.9 69.9	2.5 0.571	0.711–8.784 0.345–0.947	*P* > 0.05 Ref
Mosquitoes (presence)	Yes No	22 23	9.1 30.4	90.9 69.9	2.5 0.571	0.711–8.784 0.345–0.947	*P* > 0.05 Ref

Ab^−^ = antibody-positive; Ab^+^; OR = odds ratio; CI = confidence interval, NA = not applicable, Ref = reference category

In birds, age and breed were the two risk factors that were shown to contribute significantly (*P* < 0.05) to JEV exposure. All other factors evaluated were not statistically different (Table 4).

**Table 4:** Risk factors for Japanese encephalitis virus (JEV) seropositivity in birds

Risk factors		*n*	JEV Ab^−^ (%)	JEV Ab^+^ (%)	OR	95% CI	*P* value
Bird (*n* = 45)							
Source	Same area Same district Epidemic areas	23 12 10	78.3 50.0 80.0	21.7 50.0 20.0	1.601 NA NA	0.433–1.501 NA NA	*P* > 0.05 Ref Ref
Gender	Male Female	33 12	58.3 75.8	41.7 24.2	1.758 0.788	0.680–4.544 0.494–1.257	*P* > 0.05 Ref
Age	Young Adult	16 29	50.0 82.8	50.0 17.2	2.462 0.513	1.177–5.150 0.251–1.049	*P* = 0.020 Ref
Breed	Local Import	26 19	57.7 89.5	42.3 10.5	1.805 0.290	1.168–2.791 0.078–1.079	*P* = 0.020 Ref
Healthy status	Sick Healthy	4 41	75.0 70.0	25.0 29.3	0.821 1.019	0.094–7.182 0.840–1.235	*P* > 0.05 Ref
Owner	Shelter Personal	– 45	NA 71.1	NA 28.9	NA NA	NA NA	Ref Ref
Collection area							
Locality	Selangor Perak	22 23	63.6 78.3	21.7 36.4	1.407 0.684	0.322–1.451 0.786–2.518	*P* > 0.05 Ref
	Urban Rural	10 35	63.6 78.3	21.7 36.4	1.407 0.684	0.322–1.451 0.786–2.518	*P* > 0.05 Ref
Stagnant water	Yes No	33 12	78.8 50.0	21.2 50.0	0.663 2.462	0.390–1.126 0.971–6.239	*P* > 0.05 Ref
Paddy field	Yes No	23 22	78.3 63.6	21.7 36.4	0.684 1.407	0.322–1.451 0.786–2.518	*P* > 0.05 Ref
Ardeid birds (presence)	Yes No	39 6	74.4 50.0	25.6 50.0	0.849 2.462	0.618–1.166 0.569–10.650	*P* > 0.05 Ref
Mosquitoes (presence)	Yes No	45 –	71.7 NA	28.9 NA	NA NA	NA NA	Ref Ref

Ab^−^ = antibody-positive; Ab^+^; OR = odds ratio; CI = confidence interval, NA = not applicable, Ref = reference category

In the pig, only age and ownership status significantly influenced the risk of JEV exposure. Other factors evaluated did not contribute significantly to JEV in this species (Table 5).

**Table 5:** Risk factors for Japanese encephalitis virus (JEV) seropositivity in pigs

Risk factors		*n*	JEV Ab^−^ (%)	JEV Ab^+^ (%)	OR	95% CI	*P* value
Pig (*n* = 90)							
Source	Same area Same state	45 45	53.3 57.8	46.7 42.2	1.094 0.913	0.724–1.652 0.6–1.391	*P* > 0.05 Ref
Gender	Male Female	70 20	58.6 45.0	41.4 55	0.884 1.528	0.702–1.114 0.703–3.322	*P* > 0.05 Ref
Age	Young Adult	40 50	40.0 75.0	60.0 25.0	1.875 0.417	1.277–2.752 0.233–0.746	*P* = 0.001 Ref
Breed	Local Import	88 2	56.8 0.0	43.2 100.0	0.950 NA	0.885–1.020 NA	*P* > 0.05 Ref
Healthy status	Sick Healthy	9 81	77.8 53.1	22.2 46.9	0.357 1.105	0.78–1.626 0.968–1.261	*P* > 0.05 Ref
Owner	Shelter Personal	3 87	0.0 57.5	100 42.5	0.925 NA	0.847–1.010 NA	*P* = 0.049 Ref
Collection area							
Locality	Selangor Perak	45 45	53.3 57.8	42.2 46.7	1.094 0.913	0.723–1.652 0.6–1.391	*P* > 0.05 Ref
	Urban Rural	88 2	0.0 56.8	100 0.0	0.885 NA	0.885–1.020 NA	*P* > 0.05 Ref
Stagnant water	Yes No	89 1	100 56.2	100.0 0.0	0.975 NA	0.928–1.025 NA	*P* > 0.05 Ref
Paddy field	Yes No	- 90	NA 44.4	NA 55.6	NA NA	NA NA	Ref Ref
Ardeid birds (presence)	Yes No	88 2	54.5 100	45.5 0.0	1.042 NA	0.984–1.102 NA	*P* > 0.05 Ref
Mosquitoes (presence)	Yes No	88 2	56.8 0.0	43.2 100	0.950 NA	0.885–1.020 NA	*P* > 0.05 Ref

Ab^−^ = antibody-positive; Ab^+^; OR = odds ratio; CI = confidence interval, Central = NA = not applicable, Ref = reference category

In the cattle, gender, health status, age, breed, and source all showed a significant (*P* < 0.05) influence on JEV exposure in this species. Other risk factors assessed did not significantly affect the exposure of this animal to JEV (Table 6). Female cattle were two times more likely to test positive when compared to male cattle. Most of the female cattle were pregnant during sample collection. Sick cattle had five times more seropositive rate than healthy ones. The age of the cattle was significantly associated with JEV. Seropositive response to JEV was three times higher in young cattle. The breed types of cattle were significantly related to JEV. Imported breeds of cattle showed four times higher rate than local breed. However, when compared to the source of cattle, those imported from epidemic countries were more seropositive than those from the same state. Most of the cattle were imported from Australian and New Zealand.

**Table 6:** Risk factors for Japanese encephalitis virus (JEV) seropositivity in cattle

Risk factors		*n*	JEV Ab^−^ (%)	JEV Ab^+^ (%)	OR	95% CI	*P* value
Cattle (*n* = 90)							
Source	Epidemic area Same state	61 29	59.0 86.2	41.0 13.8	1.461 0.337	1.132–1.885 0.129–0.878	*P* = 0.010 Ref
Gender	Male Female	50 40	80.0 52.5	20.0 47.5	0.526 1.903	0.308–0.897 1.231–.2942	*P* = 0.006 Ref
Age	Young Adult	19 71	42.1 74.6	57.9 25.4	2.892 0.714	1.304–6.413 0.529–0.965	*P* = 0.007 Ref
Breed	Local Import	68 22	77.9 36.4	22.1 63.6	0.595 3.681	0.413–0.857 1.743–7.775	*P* = 0.000 Ref
Healthy status	Sick Healthy	10 80	30.0 72.5	70.0 27.5	4.908 0.789	1.367–17.621 0.645–0.987	*P* = 0.007 Ref
Owner	Shelter Personal	88 2	50.0 68.2	50.0 31.8	2.103 0.982	0.136–32.457 0.910–1.059	*P* > 0.05 Ref
Collection area							
Locality	Selangor Perak	90 –	67.8 NA	32.3 NA	NA NA	NA NA	Ref Ref
	Urban Rural	4 86	75.0 67.4	32.6 25.0	0.701 1.015	0.076–6.453 0.929–1.110	*P* > 0.05 Ref
Stagnant water	Yes No	86 4	67.4 75.0	32.6 25.0	1.015 0.701	0.929–1.110 0.076–6.453	*P* > 0.05 Ref
Paddy field	Yes No	– 90	– 67.8	– 32.2	NA NA	NA NA	Ref Ref
Ardeid birds (presence)	Yes No	86 4	67.8 75.0	32.6 25	1.015 0.701	0.929–1.110 0.076–6.453	*P* > 0.05 Ref
Mosquitoes (presence)	Yes No	90 –	67.8 NA	32.2 NA	NA NA	NA NA	Ref Ref

Ab^−^ = antibody-positive; Ab^+^; OR = odds ratio; CI = confidence interval, NA = not applicable, Ref = reference category

In the monkey, age, stagnant water, and present of ardeid birds were observed to the factors that significantly (*P* < 0.05) contributed to JEV exposure (Table 7). All other factors did not significantly influence JEV exposure in this species.

**Table 7:** Risk factors for Japanese encephalitis virus (JEV) seropositivity in monkeys

Risk factors		*n*	JEV Ab^−^ (%)	JEV Ab^+^ (%)	OR	95% CI	*P* value
Monkey (*n* = 56)							
Source	Same area Same district	53 3	84.9 100	15.1 0.0	1.067 NA	0.992–1.147 NA	*P* > 0.05 Ref
Gender	Male Female	29 27	84.9 100	15.1 0.0	1.067 NA	0.992–1.147 NA	*P* > 0.05 Ref
Age	Young Adult	29 27	75.9 96.3	24.7 3.7	0.231 1.909	0.036–1.470 1.275–2.859	*P* = 0.029 Ref
Breed	Local Import	56 -	85.7 NA	14.3 NA	NA NA	NA NA	Ref Ref
Healthy status	Sick Healthy	3 53	66.7 86.8	33.3 13.2	3.0 0.913	0.307–29.35 0.98–1.194	*P* > 0.05 Ref
Owner	Not owned	56	85.7	14.3	NA	NA	Ref
Collection area							
Locality	Pahang Perlis Kelantan	19 22 15	94.7 77.3 86.7	5.3 22.7 13.3	1.121 NA NA	1.202–2.455 NA NA	*P* > 0.05 Ref Ref
	Forest Rural	22 34	77.3 91.2	22.7 8.8	1.765 0.581	0.913–3.410 0.232–1.455	*P* > 0.05 Ref
Stagnant water	Yes No	29 27	72.4 100.0	27.6 0.0	2.286 –	1.658–3.150 –	*P* = 0.003 Ref
Paddy field	Yes No	6 50	83.3 86.0	16.7 14.0	1.20 0.977	0.160–8.977 0.739–1.291	*P* > 0.05 Ref
Ardeid birds (presence)	Yes No	34 22	76.5 100	23.5 0.0	1.846 –	1.423–2.395 –	*P* = 0.014 Ref
Mosquitoes (presence)	Yes No	56 –	85.7 NA	14.3 NA	NA NA	NA NA	Ref Ref

Ab^−^ = antibody-positive; Ab^+^; OR = odds ratio; CI = confidence interval, NA = not applicable, Ref = reference category

### Risk factors

**Fig. 2:**
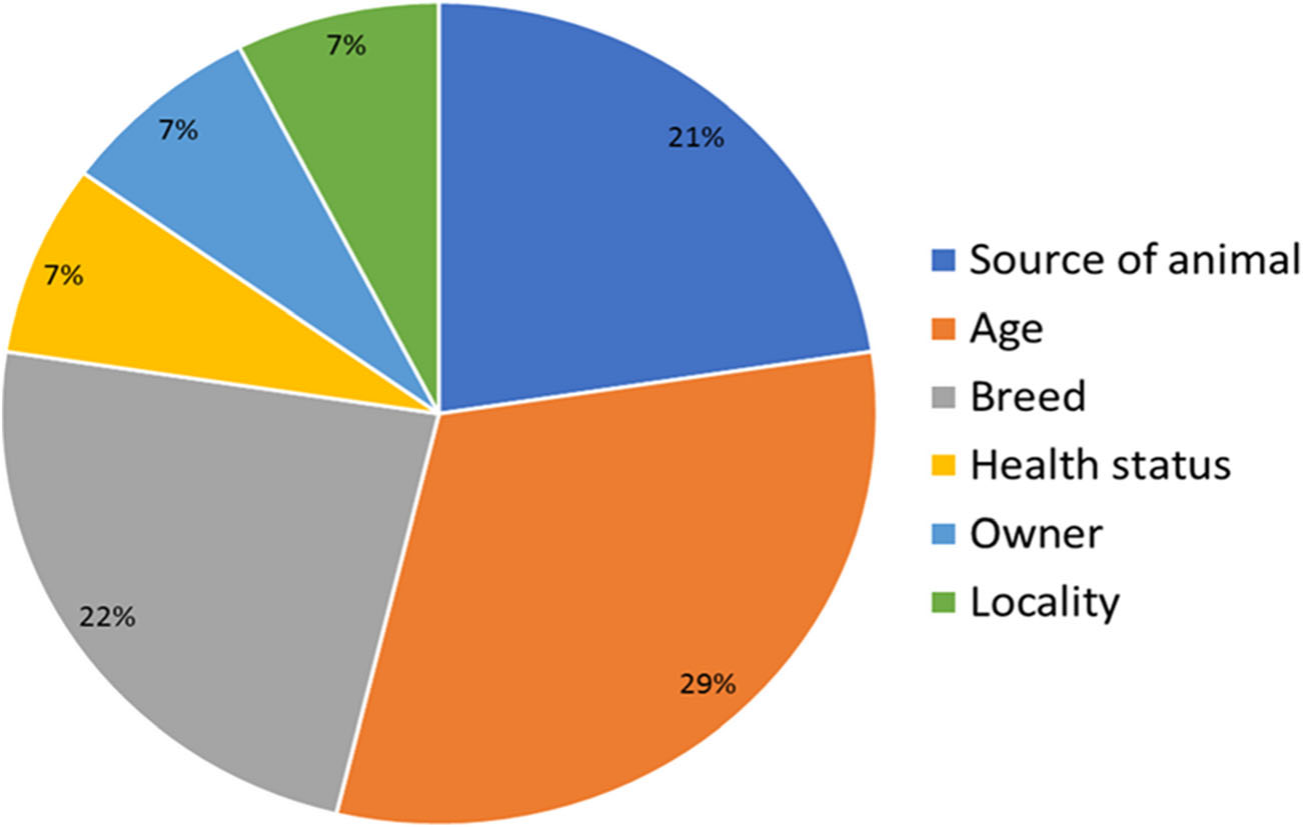
Overall risk factors of JEV infections among animals in Malaysia

In this study, 13 factors were chosen to determine the risk of JEV infection among animals in Malaysia. The most significant risk factors in JEV infections are age, breed, and source of animal (Fig. 2). This finding is similar to that shown by Thakur et al. (2012) in pigs in Nepal, where breed and animal source were the main determinant of susceptibility to JEV infections. Other minor risk factors to the infection are presence of stagnant water, mosquitoes, ardeid birds, paddy fields, and locality.

## Discussion

The high seroprevalence rate obtained in dogs in in this study was similar to the result reported in Japan (Shimoda et al. 2010, pp. 1137–1139). This is probably due to the preference and selective feeding pattern of *Culex* spp. in dogs. Some studies have suggested that the vector *Culex* spp. prefers to feed on dogs than cats or human, thus making the dogs more prone to infection (Mackenzie et al. 2006, pp. 201–268; Shimoda et al. 2010, pp. 1137–1139). This might have attributed to the high seropositive rate in dogs as compared to other animal species. The dog populations sampled in this study were from shelters where mosquitoes are likely to strive freely and feed on the dogs. The presence of caves with bat population in close proximity to the shelters in Perak state might also increase the chances of infection in the sampled dog population. A previous study demonstrated that JEV could be transmitted from mosquito-bat-mosquito at room temperature and at 10 °C (Mackenzie et al. 2006, pp. 201–268). This finding supports the possibility that bats present around the caves may result in a high potential of JEV maintenance in the environment. The susceptibility of bats to JEV has been documented previously where the virus antigen was detected in bats in India (Mackenzie et al. 2006, pp. 201–268). Bats that belonged to the Orders *Microchiroptera* and *Megachiroptera* have great potentials for the transmission and maintenance of JEV in the environment (Mackenzie et al. 2008, pp. 382–406).

Pig showed the second highest prevalence rate (44.4%). This is considered a lower prevalence rate in comparison to dogs. There are several factors that can contribute to the prevalence rate of JEV among pigs in Malaysia. Pig farms in Malaysia are normally far away from residential areas and situated in rural areas which are not close to paddy cultivation areas. The chances of JEV transmission through *Culex* spp. to pigs can be decreased due to the lack of paddy cultivation areas around pig farms in Malaysia. *Culex* spp. mainly breed in paddy areas around rural areas (Cappelle et al. 2016, pp. e0005149). They are characterized as zoophilic species also considered as primary vector for JEV (Kuwata et al. 2015, pp. 222–229). Apart from paddy cultivation areas, most of the pig farms in Malaysia practice intensive husbandry and some high-tech farms use air-conditioning to prevent stress. This makes transmission of JEV from vector to pig drastically lower. Another reason is that most of the young pigs (before 6 months) are sent for local or international consumption. Farmers never kept the pigs for more than a year and this means that old pigs are always being replaced with new pigs every few months. This makes the chances of other pigs getting infected by mosquitoes or oronasal secretions significantly reduced (Ricklin et al. 2016, pp. 10,832).

Cattle had a JEV seroprevalence rate of 32.2%. This is considered high since all cattle samples were collected from one state (Selangor). Due to the semi-intensive nature of cattle husbandry in Malaysia, cattle exposure to *Culex* spp. is very common in certain areas. Bovine are dead-end hosts for JEV (Carey et al. 1969, pp. 282–289). *C. tritaeniorhynchus* is more attracted to bovine species, hence making them high potential hosts for surveillance studies (Mackenzie et al. 2006, pp. 201–268). Countries like Sri Lanka predicted JEV infection in humans by determining the percentage of seroprevalence among cattle, goats, and pigs in the country (Peiris et al. 1993, pp. 541–548). Zoonotic transmission of JEV from cows and pigs to humans through *C. tritaeniorhynchus* and *C. gelidus* has been reported in Malaysia (Mackenzie et al. 2006, pp. 201–268). At the same time, previous research has shown that *C. tritaeniorhynchus* feed primarily on bovine followed by swine (Mitchell et al. 1973, pp. 293–299). However, *C. annulus* prefers to feed on swine followed by bovine at 2–77% feed rate. In dogs, a feed rate of 15% was reported (Mitchell et al. 1973, pp. 293–299).

Seroprevalence rate in birds was only 28.9%. This is a rather unexpected finding as the sampling area in the state is an area with high development of rice paddies. There is also a migratory bird landing areas there (Kuala Gula, Perak). There is no pig farming around the area, the residents normally engaged in fishing activities. The presence of numerous water bodies serves as suitable breeding sites for mosquitoes. A low-incidence case of JEV was previously reported in rice growing area in Thanjavur district in Tamil Nadu, India. This was attributed to the ratio of cattle to pigs in the district (Vijayarani and Gajanana 2000, pp. 212–214). This explains the reason why seropositive birds in Tanjong Piandang, Perak were low even though there were many water bodies and active rice production fields but lacked the amplifying agent, pig. In this study, village chickens showed lower seropositive rate compared to water birds. The water birds used in this study belong to Ardeidae family; previously, Ardeidae birds were reported to serve as amplifying hosts for JEV (Bhattacharya and Basu 2014, pp. 32–37). Although chickens are rarely infected with JEV and have a limited role in its transmission, a mosquito transmission study indicated that chickens were susceptible to JEV infection (Mackenzie et al. 2008, pp. 382–406).

In this study, cats show JEV seroprevalence rate of 14.4%. The role and the transmission mode of JEV among cats are still unidentified. However, some studies indicate that cats play a minimum role in the transmission cycle of JEV and show low susceptibility for JEV infection (Shimoda et al., 2010, pp. 1137–1139; Truong et al., 2013, pp. 1647–1650).

The seroprevalence of JEV in monkey was found to be 20.45%. This positive detection rate is consistent with other studies on detection of JEV antibodies. Based on the previous studies in Manila, *M. fascicularis* was shown to be naturally infected by JEV with consistent antibody prevalence rate of 35.2% (Inoue et al. 2003, pp. 89–94). Moreover, another serosurvey in Japanese macaque (*Macaca fuscata*) revealed a prevalence rate of 44% (Shimoda et al. 2014, pp. 441–445). Recently, nine out of 38 captive monkeys (*Macaca nemestrina*) colonies in northern Thailand were reported to be seropositive for JE (Nakgoi et al. 2014, pp. 97–102). Based on this study, Kelantan showed the highest prevalence of JEV as compared to Pahang and Perlis. JEV outbreak is associated with climate which favors an epidemic. Most incidences of JEV occur in the rainy season due to the formation of enlarged surface water covers that support the mass breeding of mosquito vector (Yang et al. 2004, pp. 197–205). Probably, the flooding experienced in the state of Kelantan in 2015 might have increased the risk of JE transmission, hence the high prevalence rate observed in the present study.

### Risk factors

In this study, 13 factors were used to analyze JEV exposure among animals in Malaysia. The univariate analysis showed associated between animals and different risk factors. The most significant risk factors associated with JEV infection among animals include source of animals, breed type, and age. Thakur et al. (2012) acknowledged that breed types and source of animals are significant relatives in JEV infection among pigs in Nepal. Some other factors like presence of stagnant water, paddy cultivation, presence of ardeid birds, location, nature of sampling areas, ownership, and presence of mosquitoes showed no significant relativity with JEV infection among these animals.

In this study, only source of dog showed significant association with seropositivity for JEV. This was because dogs from the same district showed twice the rate of seroposititvity as compared with other areas. All the dog samples were collected from a shelter in Selangor and Perak. Since a district can be more than one area, this can significantly contribute to the transmission of JEV.

In this study, piglet showed two times more exposure as compared with adults. Cappelle et al. (2016) reported that 28 out of 29 pigs had seroconverted before attaining the age of 6 months. This indicates that piglets have more chances to seroconvert. As previously stated, the high turnover rate of piglets as pork in Malaysian farms results in inconclusive data in the adults which are mostly breeders.

Cattle showed significant exposure with age since calves had four times more exposure to JEV than adult cattle. This may be due to maternal antibodies transmitted to the calves from the cow through colostrum. Health status was also an important factor as sick animals were seven times more exposed than healthy ones. In a previous study, Kako et al. (2014) reported that JEV infected cows showed reduced appetite, fever for 4 days, and ataxias, with clinical signs such as diarrhea also observed. Most of the imported breed of cattle showed seropositivity to JEV antibodies. Although we cannot ascertain if the source of JEV exposure was local or from the imported countries like Australia, there are records of JEV in Australia (Mackenzie et al. 2006, pp. 201–268). Cattle from epidemic areas with the sampling region were more exposed that other areas due to unknown factors. Since the cattle are bought from several farms, there is a higher chance of exposure the JEV from the other farms in Malaysia. The significant relationship between gender of cattle and JEV infection is still unclear because the two groups have different numbers of sick, young and old, breed, and source of the cattle.

In this study, young birds showed more significant exposure to JEV than adults. Previous studies have indicated that chicks are able to develop viremia and produce antibodies in JEV-endemic regions, while adult chickens develop low viremia (Cleton et al. 2014, pp. 242–246). Similarly, breed also affected the exposure rate to JEV as local breeds appeared more susceptible than imported breeds. This might be due to endemicity of the virus in the population, hence resulting in constant stimulation of the immune system and antibody production.

In this study, kittens had higher seropositive rate which was five times higher than adult cats. The locality of the sampled cats was significantly associated with JEV exposure. Cats from rural areas were three times more seropositive than urban area. Previously, JEV has been documented as a disease of rural areas (Li et al. 2016, pp. e0004611). This can be explained by poor wastewater management resulting in breeding of mosquitoes in sewage and drainages (Sutherst, 2004, pp. 136–173). Furthermore, agricultural activities withholding water tanks in rural areas predispose to higher chances of mosquito breeding sites in such areas (Thakur et al. 2012, pp. 393–400).

Based on the results, the presence of stagnant water showed two times more exposure risk to JEV in monkey. Paddy field and stagnant water are considered excellent sources for mosquito breeding (Service, 1989, pp. 228–229). Most areas sampled had stagnant water bodies, especially Perlis which also had a huge paddy field. In comparison, the risk factor of stagnant water with other factors associated with seropositivity in pigs in Nepal showed a positive correlation (Thakur et al. 2012, pp. 393–400). We would like to highlight here that *Culex* spp. are strong flyers that are able to fly around 12.6 km in Reisen et al. (1991, pp. 357–371) and Reisen et al. (1992, pp. 531–543). This makes the spread of JEV possible even though the breeding sites are far away. The presence of ardeid species in the sampling areas was significantly associated with seropositivity to JEV in monkeys. Ardeid species are important agent in JEV transmission cycles by acting as amplifier agent besides pigs (Mackenzie et al. 2006, pp. 201–268).

A total of416 samples consisted sera from livestock, companion animals and avian were pooled into group and screened for JEV using NS3 primers targeting NS3 region of JEV by one step RT-PCR (results not shown). However, all blood samples were found negative for JEV antigen detection. This rapid test did not detect antigen in both JEV seropositive and seronegative animals. This can be explained by the presence of low levels of viremia in the hosts which may have contributed to false negative at molecular assay (Cloherty et al. 2016, pp. 265–273). Apart from that, tonsil is the predominant site for JEV replication, perhaps oronasal swabs is a more suitable biological material to be used to detect JEV antigen in animals (Ricklin et al. 2016, pp. 10832).

Since most JEV cases were detected during natural infection, the acute phase of virus replication is often unknown. Virus antigen was not detected in blood samples taken during non-acute phase or during neutralizing activities (Casenghi et al. 2014, pp. 1–23). In addition, cross-neutralization among flaviviruses are other factors contributing to the difficulty in JEV antigen detection which include short viremia phase, speedy neutralizing antibody production, and reaction towards JEV antigen (Mansfield et al. 2011, pp. 2821–2829). There are approximately 11 types of flaviviruses circulating in Malaysia and this predisposed to the high flaviviruses serocomplex cross reaction.

This study has provided with the baseline information for the understanding of epidemiology and risk factors in JEV infection, transmission, and spread. This study demonstrated that most animals in Malaysia are susceptible to JEV infections. However, the pigs, dogs, and cattle are most susceptible to become JEV seropositive with exposure to the virus. JEV seropositivity is significantly influenced by several risk factors, where young aged animals, presence of mosquito vectors, and migrating birds had major influence on the development of infections in these animals. Evidently, the infection is highly prevalent in Peninsular Malaysia and control measures are to be in place to ensure that the disease is contained.
